# Supplementary material for: Improving Properties of Starch-Based Adhesives with Carboxylic Acids and Enzymatically Polymerized Lignosulfonates
Source: Int J Mol Sci. 2022 Nov 4;23(21):13547. doi: 10.3390/ijms232113547 (PMC9657476; doi:10.3390/ijms232113547)
Supplement: Supplementary file 1 [file ijms-23-13547-s001.zip › ijms-1918703-supplementary.pdf]

Supplementary Material

Improving properties of starch-based adhesives with carboxylic acids and enzymatically polymerized lignosulfonates.

Miguel Jimenez Bartolome [1,*], Sidhant Satya Prakash Padhi [1], Oliver Gabriel Fichtberger [1], Nikolaus Schwaiger [2], Bernhard Seidl [3], Martin Kozich [3], Gibson S. Nyanhongo [1,4] and Georg M. Guebitz [1]

[1] Institute of Environmental Biotechnology, University of Natural Resources and Life Sciences, Vienna, Konrad Lorenz Strasse 20, 3430 Tulln an der Donau, Austria
[2] Sappi Papier Holding GmbH, BruckerStrasse 21, 8101 Gratkorn, Austria
[3] Agrana Research & Innovation Center GmbH, Josef Reitherstraße 21–23, 3430 Tulln, Austria
[4] Department of Biotechnology and Food Technology, Faculty of Science, University of Johannesburg, Corner Siemert and Louisa, Doornfontein, John Orr Building, 2028 Johannesburg, South Africa
* Correspondence: miguel.jimenez-bartolome@boku.ac.at

Characterization of laccase polymerized lignosulfonates.

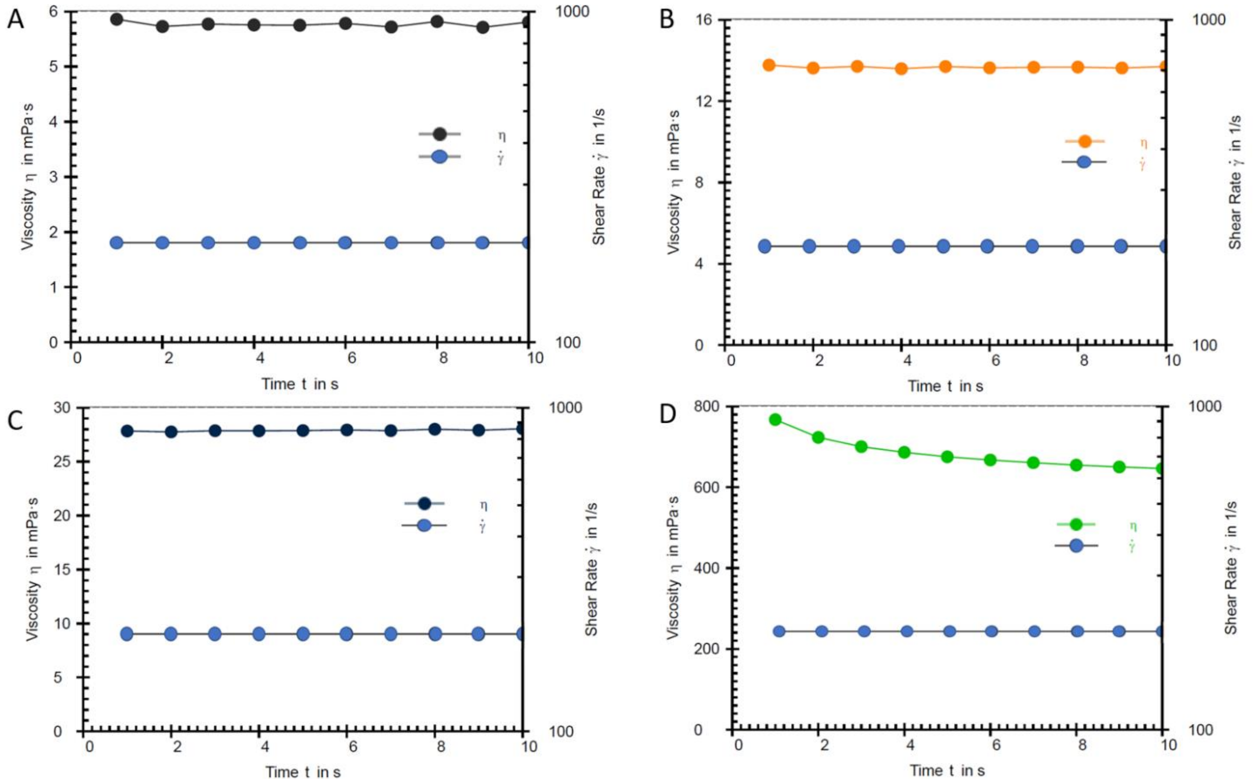

Figure S1. Viscosity curves of the different time-points of the lignosulfonate polymerization: 0 min (A), 20 min (B), 50 min (C) and 90 min (90). The viscosity is presented in mPa*s and the shear rate in 1/s. The shear rate was constant in all the measurements.

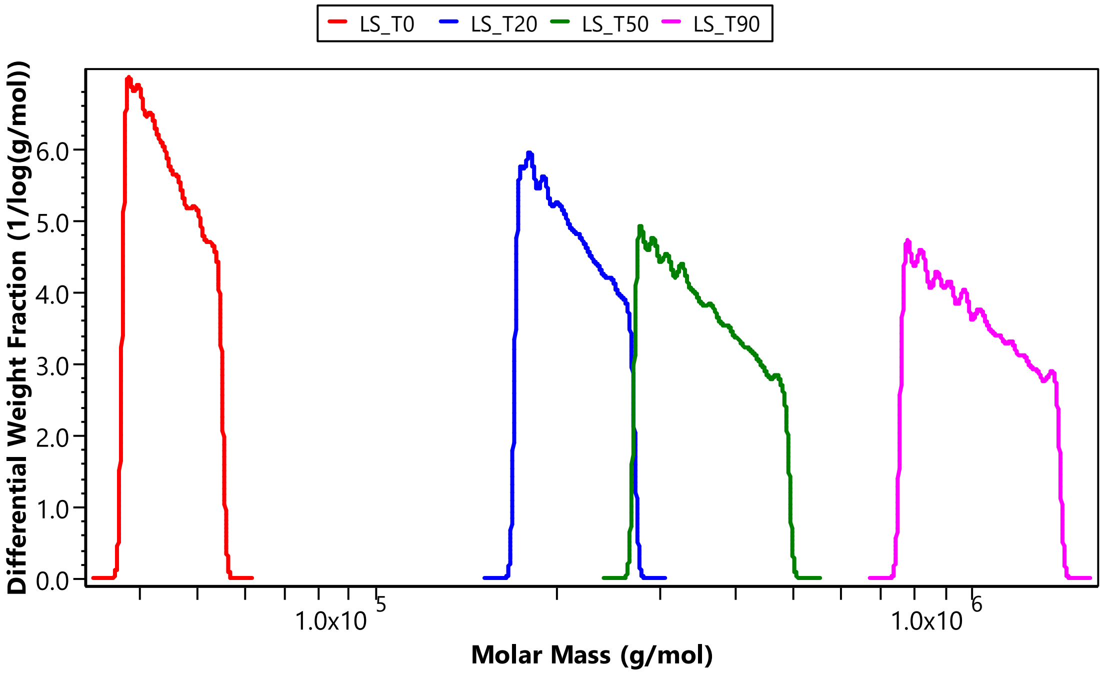

Figure S2. Molecular mass of the lignosulfonate polymerization obtained by size exclusion chromatography (SEC). In the figure, the molar masses of different time-points of the reactions are presented (from left to right: 0, 20, 50 and 90 minutes).
